# Isoniazid-Induced Acute Liver Failure Requiring Liver Transplantation

**DOI:** 10.7759/cureus.32905

**Published:** 2022-12-24

**Authors:** Mariana S Almeida, Francisco Gomes, Maria do Rosário Ginga, Martinho Fernandes, Joana Cartucho

**Affiliations:** 1 Internal Medicine, Hospital do Barreiro, Barreiro, PRT

**Keywords:** tuberculosis, liver transplantation, tuberculostatics, hepatotoxicity, acute liver failure, isoniazid

## Abstract

Tuberculosis (TB) is a contagious disease caused by the bacilli *Mycobacterium tuberculosis* complex. Currently, about a quarter of the world's population is infected with *Mycobacterium tuberculosis*. According to the World Health Organization, in 2018, Portugal had a TB incidence rate of about 24 cases per 100,000 inhabitants. It is estimated that 5-15% of individuals with latent TB infection progress to active TB. Isoniazid is one of the most widely used drugs for the treatment of active and latent TB. However, care must be taken with the possible development of toxicity, particularly hepatic.

The authors report a case of a 60-year-old woman diagnosed with latent TB who started therapy with isoniazid. Five months later, she developed acute liver failure secondary to the drug, requiring liver transplantation, with a favorable clinical outcome. Thus, we intend to alert to the potential toxicities of isoniazid, establishing follow-up strategies in patients on this therapy.

## Introduction

The vast majority of cases of tuberculosis (TB) in countries with low rates of the disease are caused by the reactivation of latent infection. Therefore, treating the latent infection is essential to curb TB cases.

Several anti-TB drug regimens exist for the treatment of TB and latent tuberculosis infection (LTBI), most commonly including the use of isoniazid or isonicotinic acid [1]. Hepatotoxicity associated with tuberculostatics is well-recognized as one type of drug-induced liver injury (DILI).

We describe a case of a 60-year-old woman who had been taking isoniazid (INH) and pyridoxine for about five months in the context of latent infection. No other relevant history was present. She was referred to the emergency service for a 15-day evolution of nausea, abdominal pain in the upper quadrants, and progressive jaundice that led to hospitalization. Subsequently, she developed acute hepatic failure with coagulopathy and hepatic encephalopathy, requiring orotracheal intubation and sedation. She was transferred to a liver transplantation unit, where she underwent a successful liver transplant. Her clinical evolution was favorable, and she was discharged.

The authors consider this case relevant since the diagnosis of acute liver failure is highly specific and rare, characterized by analytical changes in the liver profile in an individual without previous liver disease. However, it is often confused with the worsening of chronic liver disease, making diagnosis more difficult. It is also important to be alert to the possible etiologies, not forgetting the pharmacological history, which in this case allowed a timely diagnosis and was crucial for the survival of the patient, who quickly underwent a liver transplant.

## Case presentation

**Figure 1:**
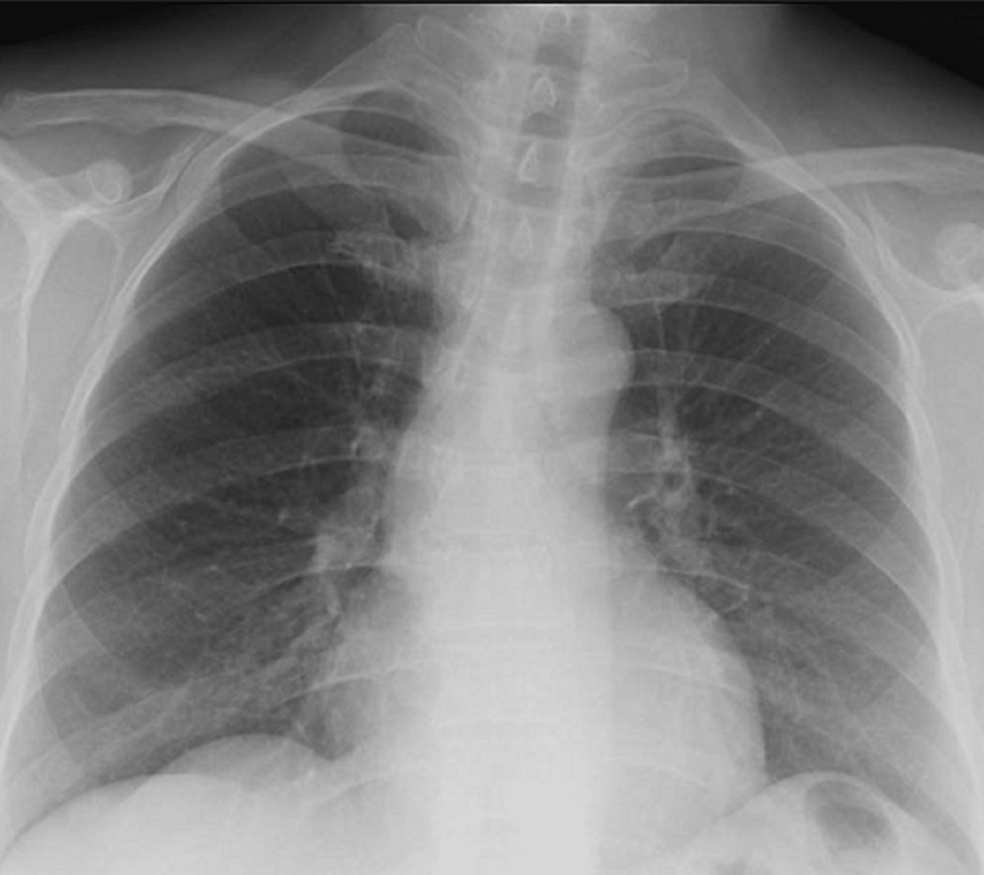
Chest X-ray

A 60-year-old woman, an operational assistant at our hospital, with no personal history, regular medication, or allergies, had contact with a patient infected with TB at the hospital and was referred for a TB appointment in April. She denied consumption of alcohol or other toxins, teas, or herbal products. She was asymptomatic and underwent tests, with a positive interferon-gamma release assay (IGRA) and positive Mantoux test with 14 mm, with an unchanged chest X-ray, thus admitting the diagnosis of latent TB (Figure 1).

Other diagnostic or antibacillary resistance tests were not performed, in particular, the GeneXpert assay. Thus, she started therapy with isoniazid at a daily dose of 300 mg and pyridoxine and was followed up in a medical appointment with no intercurrences.

After five months, she went to the hospital for 15 days of evolution of abdominal pain in the upper quadrants, nausea, and jaundice. She also reported progressive fatigue from efforts, anorexia, pale stool, and dark urine. She was conscious and oriented, with jaundice of the skin and sclerae. No palpable adenopathies or masses were noted. The abdomen was slightly painful on palpation of the upper quadrants, without peritoneal irritation, and no hepatomegaly or splenomegaly was noted.

Blood tests showed normal blood cell count, without changes in renal function or infection parameters, with an increased international normalized ratio (INR) of 3.33. The liver profile also showed alterations, with an increased cholestatic pattern with aspartate aminotransferase (AST) at 1569 U/L, alanine aminotransferase (ALT) at 1571 U/L, alkaline phosphatase (AP) at 243 U/L, total bilirubin at 15.9 mg/dl, and direct bilirubin at 8.7 mg/dl (Table 1).

**Table 1:** Laboratory tests AST: aspartate aminotransferase; ALT: alanine aminotransferase; AP: alkaline phosphatase; HT: hepatic transplantation; INR: international normalized ratio; MELD: Model for End-stage Liver Disease; Na: sodium.

Laboratory tests	Day 1 (admission)	Day 5	Day 6	Day 9	2 days after HT	4 months after discharge
Total bilirubin (0.1-1.0 mg/dl)	15.9	21	18.7	16.7	-	0.41
Direct bilirubin (0-0.3 mg/dl)	8.7	11.2	9.6	7.86	-	-
AST (15-40 U/L)	2569	1928	1380	387	-	33
ALT (10-40 U/L)	1571	1207	1026	504	-	36
AP (30-115 U/L)	234	238	218	141	-	57
Albumin (3.5-5.5 g/dl)	2.6	-	2.9	2.3	-	-
Sodium (135-146 mmol/L)	135	136	136	145	-	141
Creatinine (0.6-1.2 mg/dL)	0.93	0.74	0.79	0.7	-	0.9
INR (0.8-1)	3.33	2.94	3.06	3.2	-	0.96
Prothrombin time (<12 sec)	38	34.7	36.1	-	10.8	10.6
MELD-Na	32	30	31	30	-	-

An abdominal ultrasound was requested, describing a “liver with bosselated contours, structural heterogeneity, and hepatic atrophy." Thus, the diagnostic hypothesis of hepatitis of unexplained etiology was put forward.

Further work-up was carried out, excluding Wilson's disease, hemochromatosis, alpha-1-antitrypsin deficit, primary biliary cholangitis, primary sclerosing cholangitis, and viral hepatitis (Table 2).

**Table 2:** Laboratory tests for the etiological study of acute liver failure Anti-LKM: liver kidney microsome type 1 antibody; AMA: antimitochondrial M antibody; ANA: antinuclear antibody; anti-dsDNA: double-stranded deoxyribonucleic acid antibody; ANA: antinuclear antibody; HBV: hepatitis B virus; HAV: hepatitis A virus; HCV: hepatitis C virus; CMV: cytomegalovirus; HIV: human immunodeficiency virus; EBV: Epstein-Barr virus; IgG: immunoglobulin G; IgM: immunoglobulin M.

Etiological study	Results
Iron (50-150 ug/dL)	211
Total iron-binding capacity (250-450 ug/dL)	<25
Ferritin (20-150 ng/mL)	4247
Transferrin (215-380 mg/dL)	160
Haptoglobin (25-190 mg/dL)	<8
Alpha-1-antitrypsin (200-400 mg/dL)	134
Ceruloplasmin (21-53 mg/dL)	27
Anti-LKM, AMA, anti-dsDNA, ANA	Negative
Serologies for HBV, HCV, HAV, CMV, HIV, and EBV	Negative
IgG (650-1640 mg/dL)	1564
IgM (40-230 mg/dL)	276

Acetaminophen doses were checked despite no history of paracetamol use, with a negative result. The patient had no alcoholic habits, so this etiology was excluded. Thus, the possibility of a DILI secondary to isoniazid was raised and the drug was suspended.

**Figure 2:**
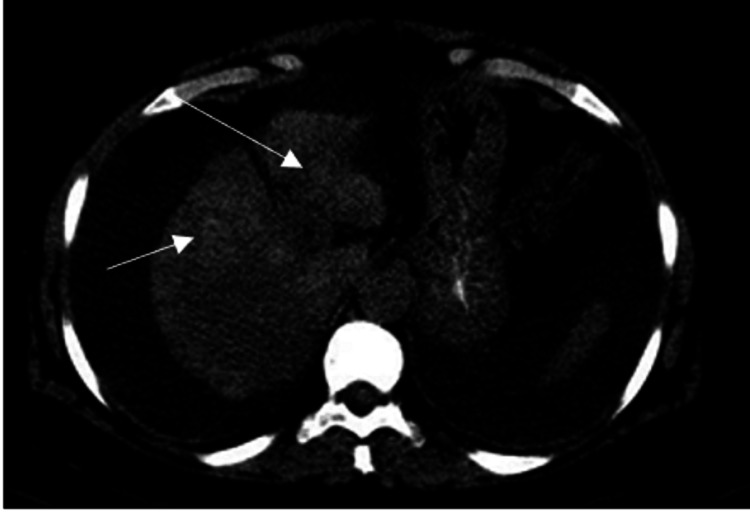
CT scan showing hepatic atrophy

Five days after admission, due to the maintenance of the elevated cholestatic pattern, and still without autoimmunity results, it was decided to start corticoid therapy with prednisolone 60 mg/day, admitting a possible autoimmune etiology. Abdominopelvic CT was also requested with images compatible with hepatic atrophy, with no other alterations (Figure 2). By this time, the patient remained conscious and oriented, with skin jaundice, without flapping, and with an innocent abdomen.

With the institution of corticotherapy, there was an improvement in the cholestatic pattern. However, two days later, the patient presented mental confusion and hallucination, with the hypothesis of being a psychosis associated with the corticoids. Therefore, they were suspended. The patient also started a laxative, both with no improvement.

Brain computed tomography was done, revealing "discrete subcortical frontal and parietal hypodensities of greater expression to the left, of possible microangiopathic vascular etiology."

By this time, eight days after admission, due to worsening of the neurological state, she was transferred to our intermediate unit, where orotracheal intubation was performed for airway protection.

The hypothesis of a portosystemic encephalopathy was also put forward, with grade III on the West Haven scale. With the most likely diagnostic hypothesis of acute hepatic failure secondary to isoniazid, contact was made with the liver transplantation unit, which accepted the patient. She was submitted to an isograft orthotopic liver transplant using the piggyback technique with a T-tube with a duration of around five hours, without complications. After surgery, the patient was successfully extubated one day later.

A biopsy of the specimen of total hepatectomy macroscopically highlighted "irregular capsular surface" and microscopically highlighted "extensive confluent necrosis and inflammatory infiltrate," confirming the diagnosis of massive hepatic necrosis, in a context of toxic etiology. The final diagnosis of acute hepatic failure secondary to isoniazid was accepted. The remaining hospital stay was uneventful, with a favorable clinical and analytical evolution.

## Discussion

Isoniazid is arguably among the most clinically successful and extensively studied TB drugs ever to have been developed. It is a bactericide that inhibits the synthesis of mycolic acids in the bacterial cell wall, and it is effective against intracellular and extracellular organisms. INH has been approved for inclusion in combination therapies for active infection and has also been approved as a prophylactic monotherapy to prevent disease in individuals with an asymptomatic TB or LTBI [2].

By 2020, the guidelines for the treatment of LTBI recommend rifamycin-based regimens; six or nine months of daily isoniazid are alternative recommended regimens; although efficacious, they have higher toxicity risk and lower treatment completion rates, which decrease effectiveness [3].

Despite INH’s proven and robust efficacy, it has long been recognized as hepatotoxic and can cause liver failure [2]. In about 1% of patients, serious hepatotoxicity develops, characterized by plasma aminotransferase activities of >5 × ULN, and very rarely fulminant liver failure. These patients present with symptoms including abdominal pain, nausea, vomiting, and jaundice, as was seen in our patient [4].

A recent literature review of cases from 1994 to 2015 identified at least eight other cases of fulminant hepatic failure requiring liver transplantation that was attributed to INH, individually or in combination with other drugs [1].

The Centers for Disease Control and Prevention published a report that quantified the frequency of severe adverse effects in patients receiving isoniazid for LTBI treatment during 2004-2008. Severe liver injury due to isoniazid was reported in 17 patients, five of whom underwent liver transplantation. Five patients died, including one patient who had liver transplantation [5].

Considering jaundice as the first symptom, hyperacute liver failure describes patients developing hepatic encephalopathy (HE) within seven days of noting jaundice. Acute liver failure occurs when patients develop HE between eight and 28 days of noting jaundice, and subacute liver failure describes HE occurring within five to 12 weeks of jaundice. Disease duration of greater than 28 weeks before the onset of encephalopathy is categorized as a chronic liver disease [6].

In this clinical case, the patient developed acute liver failure about 20 days after the onset of jaundice, so this is a case of acute liver failure.

Various agents can result in hepatocyte or bile duct injury, or both, with a pattern that is hepatocellular, cholestatic, or mixed. INH-induced hepatotoxicity manifests mainly as hepatocellular necrosis, as seen in the present clinical case. Medications can cause liver injury in a predictable time and dose-dependent manner (such as high doses of acetaminophen), whereas others such as INH do so more unpredictably or in an “idiosyncratic” manner [2].

Known risk factors that make an individual susceptible to INH hepatotoxicity include advanced age, female gender, alcohol use, cirrhosis or other pre-existing liver diseases, having an Asian racial background, chronic viral hepatitis, and concurrent use of other known potential hepatotoxic medications [1]. Current studies have also shown that differences in the rate of INH-induced hepatotoxicity in individuals can be attributed to genetic variability at several loci that code for drug-metabolizing enzymes; for example, the slow acetylator status of N-acetyltransferase 2 and cytochrome P450 2E1 C/D or C/C genotype together are associated with a higher frequency of hepatotoxicity [4].

INH-induced hepatotoxicity occurs with variable latency, with a latency period of approximately one week to three months in most cases, although the disease can occur up to one year later or more [4]. In the present clinical case, the patient presented hepatotoxicity after five months of treatment.

Li et al. reported a similar case of a 67-year-old Indian male who had been on isoniazid and pyridoxine therapy for three months for latent TB, developed acute liver failure, and underwent liver transplantation about six weeks after the onset of symptoms [3].

Khan et al. reported a 42-year-old Indian male who received rifampicin and isoniazid daily after being diagnosed with LTBI. He developed fulminant hepatic failure secondary to antituberculosis drugs and was transferred immediately to the medical intensive care unit, where he died four days later [7].

Some experts recommend that INH be withheld if a patient’s transaminase level exceeds three times the upper limit of normal in association with symptoms and five times the upper limit of normal if the patient is asymptomatic.

Delays in INH discontinuation may result in irreversible hepatic destruction. The prognosis of acute liver failure may be improved by liver transplantation; however, if this therapy is not an option, the prognosis of patients with acute liver failure is poor [4].

## Conclusions

We report a case of acute liver failure in a middle-aged woman taking isoniazid for five months for latent TB, despite the withdrawal of the drug, requiring liver transplantation, with a consequent favorable outcome. Discontinuation of isoniazid generally improves hepatotoxicity, so this case is a particular example of the rare occurrence of the progression to acute liver failure. The clinical symptoms were typical and the strong clinical suspicion of hepatic failure led to early contact with the transplantation unit, favorably changing the patient’s prognosis.

The authors intend to alert to the potential adverse effects of tuberculostatics, namely, isoniazid, so the patient must have very tight control to avoid potential toxicities, namely, hepatotoxicity.
